# From Waste to Treasure: Therapeutic Horizons of Polyhydroxyalkanoates in Modern Medicine

**DOI:** 10.3390/pharmaceutics18010082

**Published:** 2026-01-08

**Authors:** Farid Hajareh Haghighi, Roya Binaymotlagh, Paula Stefana Pintilei, Laura Chronopoulou, Cleofe Palocci

**Affiliations:** 1 Department of Chemistry, Sapienza University of Rome, Piazzale Aldo Moro 5, 00185 Rome, Italyroya.binaymotlagh@uniroma1.it (R.B.); paulastefana.pintilei@uniroma1.it (P.S.P.); laura.chronopoulou@uniroma1.it (L.C.); 2 Research Center for Applied Sciences to the Safeguard of Environment and Cultural Heritage (CIABC), Sapienza University of Rome, Piazzale Aldo Moro 5, 00185 Rome, Italy

**Keywords:** polyhydroxyalkanoates, biodegradable plastics, bioplastic, medical biomaterials, drug delivery, tissue engineering, wound healing, antimicrobial coatings, waste valorization, therapeutic applications

## Abstract

Polyhydroxyalkanoates (PHAs), a family of biodegradable polyesters produced through microbial fermentation of carbon-rich residues, are emerging as attractive alternatives to petroleum-based plastics. Their appeal lies in their exceptional biocompatibility, inherent biodegradability, and tunable physicochemical properties across diverse applications. These materials are environmentally friendly not just at the end of their life, but throughout their entire production–use–disposal cycle. This mini-review presents an update on the expanding biomedical relevance of PHAs, with emphasis on their utility in tissue engineering and drug delivery platforms. In addition, current clinical evaluations and regulatory frameworks are briefly discussed, underscoring the translational potential of PHAs in meeting unmet medical needs. As the healthcare sector advances toward environmentally responsible and patient-focused innovations, PHAs exemplify the convergence of waste valorization and biomedical progress, transforming discarded resources into functional materials for repair, regeneration, and healing.

## 1. Introduction

### 1.1. Traditional Plastics: Convenience at the Cost of the Planet

Plastics are among the most widely utilized materials worldwide, valued for their adaptability across sectors ranging from large-scale manufacturing to everyday consumer packaging. The most common plastics—made from polyethylene (PE), polypropylene (PP), polystyrene (PS), polyvinyl chloride (PVC), polyurethane (PUR), polyethylene terephthalate (PET), and polybutylene terephthalate (PBT)—are primarily derived from petrochemical sources (Table 1) [1,2].

Their success stems from low production costs compared to alternatives, light weight density, mechanical strength, and ease of processing, which have enabled their integration into nearly every aspect of modern economies. However, this reliance has triggered escalating environmental and socio-economic concerns [3]. Petrochemical plastics contribute significantly to greenhouse gas emissions during production and disposal, while their resistance to degradation means they persist in soil, rivers, and oceans for centuries [4]. Current estimates suggest 400 million tonnes of plastic are produced annually, with half designed for single-use applications. Alarmingly, only about 10% of this plastic is recycled, while 19–23 million tonnes leak into aquatic ecosystems each year, a figure projected to rise by 50% by 2040 if no action is taken [5,6,7,8,9]. The linear “produce–use–discard” model, especially in packaging systems, has intensified ecological damage. Plastics fragment into microplastics and nanoplastics, which are now found in virtually every ecosystem—from polar ice to coral reefs—and even in human food, water, and air [10,11,12]. The degradation process is modulated by abiotic factors such as ultraviolet (UV) radiation and mechanical weathering, as well as by biotic activity involving microorganisms [13,14]. Studies estimate that the average person ingests over 50,000 plastic particles annually, with inhalation raising this number significantly [15,16]. These particulate contaminants can be ingested and subsequently bioaccumulate within tissues, where they may induce toxicological or carcinogenic effects [17], and disrupt food chains. Several bacterial genera (*Arthrobacter*, *Pseudomonas*, *Bacillus*, *Streptomyces*, *Rhodococcus*, *Micrococcus*, *Corynebacterium*, *Nocardia*) and fungal groups (*Fusarium*, *Aspergillus*, *Penicillium*) produce extracellular enzymes capable of cleaving polymer chains into lower-molecular-weight compounds [18]. Nevertheless, evolutionary processes have not yet yielded highly specialized enzymatic systems capable of efficiently mineralizing synthetic polymers. Complicating this further, many plastics are engineered as complex composites incorporating solubilizers and chemical additives to enhance mechanical strength and physicochemical performance, thereby rendering natural biodegradation even more challenging [19].

### 1.2. Bioplastics as Sustainable Alternatives to Petroleum-Based Plastics

In recent times, attention has shifted toward finding substitutes for petroleum-based plastics [20]. A major focus has been on bio-based polymers, or bioplastics, which pose fewer risks to human health and the environment [21,22]. These materials are made entirely or partly from natural sources like rubber or latex and have a long history of use—the first man-made biopolymer, based on cellulose, was created in the 1800s [23]. Bioplastics are typically classified into two groups: (1) the first includes biodegradable types, which can break down naturally into simpler compounds; and (2) the second consists of nonbiodegradable types, which do not decompose on their own and must be processed through industrial composting. Biopolymers themselves fall into three main categories: (1) one group comes directly from biological matter, such as starches, proteins like collagen or gelatin, and fats; (2) another group is produced from renewable raw material intermediates, with polylactic acid (PLA) being a common example; and (3) the final group is generated by microorganisms, with polyhydroxyalkanoates (PHAs) considered as the most notable [24].

PHAs stand out due to their role as naturally occurring polyesters, produced in limited quantities by a wide range of prokaryotic species and, to some extent, by certain eukaryotes. Within these organisms, PHAs accumulate as intracellular granules that function as reservoirs of carbon and energy. A key advantage of these biopolymers is their complete biodegradability: microbial processes can convert them back into simple end products such as carbon dioxide and water [25]. Because of this environmentally benign breakdown, PHAs are often highlighted as promising substitutes for petroleum-derived plastics, offering the durability and versatility of conventional polymers while ensuring ecological compatibility throughout their life span. However, despite their potential, large-scale commercialization remains constrained by the high expense of production, which emphasizes the importance of ongoing scientific innovation and technological progress to make them economically viable [26].

While several recent reviews have focused primarily on PHAs as drug-delivery carriers, the present mini-review takes a broader biomedical perspective. We integrate advances in tissue engineering, emerging clinical evaluations, regulatory considerations, and sustainability-driven production strategies to highlight how PHAs uniquely bridge waste valorization with translational medical innovation.

## 2. Characteristics and Applications of PHAs

**Figure 1:**
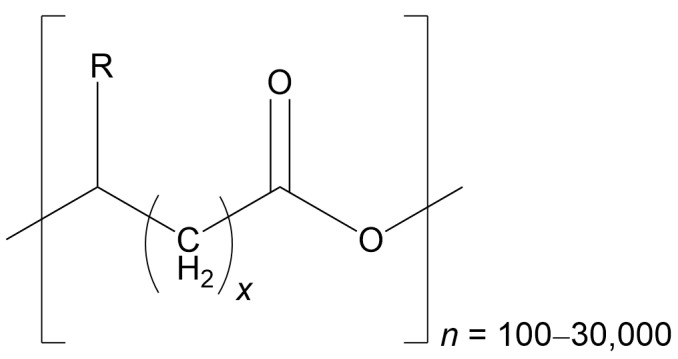
The general structure of PHAs is represented as follows: R can range from hydrogen to pentadecyl, *n* represents the number of repeats of the monomer(s) (which can be arranged in tandem or randomly), and *x* signifies the number of CH_2_ groups within the main chain of each monomer. For the classification of PHAs, the total number of carbons present in the monomer is used, calculated as (R + *x* + 2) (Redrawn from reference [1], MDPI 2024).

PHAs form a wide group of biomaterials, and their characteristics depend on both the kind and sequence of monomers that make up their polymer chains. While more than 150 hydroxyalkanoate monomers with the ability to generate PHAs have been discovered, only a limited number are currently used at an industrial scale [27]. The classification of PHAs is based on the count of carbon atoms present in their main chain or in the side group (R), as shown in Figure 1.

Biopolyesters are typically classified according to the length of their monomer chains. Short-chain PHAs (scl-PHAs) consist of 4–5 carbon atoms, medium-chain PHAs (mcl-PHAs) fall within 6–14 carbons, and long-chain PHAs (lcl-PHAs) extend beyond 14 carbons. In some research, these boundaries are expanded, with mcl-PHAs defined as having 6–16 carbons, while lcl-PHAs are described as containing more than 16 carbons. The variation in side-chain length significantly influences both the structural behavior of the polymer and its potential fields of application (Table 2) [1].

Another way to classify PHAs is by their molecular composition; homopolyesters are formed from a single monomer type, whereas heteropolyesters incorporate multiple monomers. Heteropolyesters can be further divided into subgroups: copolyesters include two monomers, terpolyesters contain three, and quaterpolyesters are built from four distinct monomers [36]. The incorporation of different monomers produces heteropolymers with properties that are intermediate between those of the individual homopolymers from which they originate.

Another way to classify PHAs is by examining the carbon sources used during their synthesis. They can be produced from traditional raw materials such as sugars, plant oils, or animal-derived fatty acids, which serve as precursors for hydroxy acid monomers [37]. In contrast, altered carbon sources may be utilized to generate polymers containing new functional groups within their side chains, leading to changes in both structure and physical performance [1]. Current studies are investigating chemical modifications—such as carboxylation, hydroxylation, and graft copolymerization—to improve traits like water resistance and compatibility with biological systems, thereby positioning PHAs as highly suitable for biomedical applications [38]. PHAs are particularly valued because of their distinctive benefits, including their ability to degrade naturally, their compatibility with living tissues, and their non-toxic nature [39,40]. These attributes make them versatile materials with applications spanning medicine, agriculture, and renewable energy production [41,42].

## 3. Waste as a Resource to Produce Bioplastics

The cost of producing polyhydroxybutyrate (PHB) is estimated to fall between 5380 and 18,300 USD per tonne of purified polymer. This high expense is largely dictated by the price of feedstock, which has prompted extensive research into utilizing agricultural byproducts and various microbial strains to enhance economic feasibility [43]. Since the agri-food industry generates vast amounts of waste—often of poor quality or environmentally damaging—these residues can be redirected into PHA production, transforming them into valuable bioplastics.

As an illustration, the cocoa sector generates about 4.2 tonnes annually, with pods making up 75% of the fruit’s weight. This translates into roughly 10 tonnes of waste for every tonne of dry beans produced. Studies have shown that *Cupriavidus necator* can accumulate PHB at 58.60 ± 4.95% of its cell dry mass when cultivated on alkaline cocoa pod hydrolysates [44]. Likewise, discarded fruits and vegetables have proven to be efficient feedstocks, allowing *Cupriavidus necator* to achieve PHA yields as high as 79.20% of cell dry mass (*w*/*w*) [45].

Cheese whey, the principal byproduct of dairy manufacturing, is composed mainly of water (over 90%), along with lactose (4.5–5.0% *w*/*v*), proteins (0.6–0.85% *w*/*v*), minor amounts of fat (0.36%), and minerals (0.53%) [46]. Microbial communities capable of generating biohydrogen while simultaneously storing PHAs from whey have shown notable efficiency [47]. Similarly, the wine industry produces around 5 tonnes of waste per hectare each year, which can be repurposed to mitigate environmental damage [48]. Strains such as *Cupriavidus necator*, *Halomonas halophila*, and *Halomonas organivorans* have demonstrated PHB accumulation rates of 70.4 ± 2.4%, 57.0 ± 1.0%, and 55.4 ± 1.4% of cell dry mass, respectively, when cultivated on grape sugar extract [49]. Other genera, including *Bacillus*, *Tepidimonas*, *Azotobacter*, and *Pseudomonas*, have also exhibited potential in converting wine-derived residues into PHAs [50].

Slaughterhouse byproducts, which are rich in fats, can be metabolized by *Cupriavidus necator* and *Pseudomonas oleovorans* to synthesize scl-PHAs [51]. Recombinant strains of *Delftia acidovorans* have been engineered to utilize substrates such as corn oil, udder, lard, and tallow, achieving yields of 26.72 ± 6.66%, 26.72 ± 6.66%, 39.33 ± 1.04%, and 15.33 ± 6.11% of cell dry mass, respectively [52]. Olive oil production, particularly concentrated in the Mediterranean region, generates approximately 2000 tonnes annually, along with more than 30 tonnes of wastewater from mills [53]. This effluent, which contains carbohydrates, lipids, and volatile fatty acids, provides a cost-effective feedstock for PHA synthesis [54]. *Bacillus amyloliquefaciens* OM81, capable of accumulating 30.2 ± 0.3% PHAs on glucose, has also been shown to utilize olive mill wastewater, producing yields between 5.6 ± 0.1% and 11.2 ± 0.3%. Additionally, waste frying oils have proven to be effective substrates for PHA production [55].

Beet molasses, a secondary product of sugar beet refining (0.25–0.35 tonnes generated per 7 tonnes of beets), can be directly applied as a substrate in fermentation processes [56]. *Cupriavidus necator* has been effectively cultivated on beet molasses, leading to the synthesis of P(3HB-co-3HV) [57]. When desugared beet molasses is used, *Bacillus megaterium* strains have achieved yields of 55–60% bioplastic per dry cell mass [58]. Additionally, species of *Parapedobacter* have demonstrated the ability to produce PHB from molasses [59].

The wood-processing sector also generates non-edible residues that can serve as low-cost raw materials for producing valuable bioproducts [60]. Among these, lignocellulose stands out as one of the most abundant renewable resources globally, arising as a major byproduct of both agriculture and forestry [61]. Although its utilization requires advanced microbial systems capable of breaking down lignin, microbial consortia have been shown to successfully convert lignocellulosic hydrolysates into PHAs [62].

**Figure 2:**
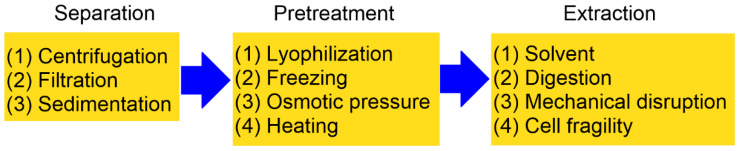
A schematic diagram illustrating the steps required to obtain PHAs generated by microorganisms (reproduced from reference [1], MDPI, 2024).

Another major factor contributing to the higher cost of PHAs compared to petroleum-derived plastics is the extraction process, which represents nearly 30% of the overall production expense [63]. Reported recovery methods are generally classified into chemical and physical techniques, which may be applied independently or in combination [2,64]. The process of isolating PHAs from fermentation cultures typically involves four essential stages: (1) biomass separation; (2) biomass pretreatment; (3) polymer extraction; and (4) purification of the final product (Figure 2). To ensure optimal yield and polymer quality, extraction is commonly carried out during the peak phase of bacterial growth [65]. Among the four stages of PHA isolation, biomass pretreatment and polymer extraction are generally considered the most labor-intensive steps. Pretreatment often requires cell disruption through mechanical (e.g., high-pressure homogenization, bead milling) or chemical means, both of which demand significant energy input and operational time. The greatest loss of biopolymer typically occurs during the extraction stage, particularly when harsh solvents or incomplete cell lysis lead to partial polymer degradation or incomplete recovery.

Several studies have proposed solutions to mitigate these challenges. Enzymatic digestion and mild surfactant-assisted treatments have been shown to reduce energy consumption while improving polymer integrity. Likewise, non-chlorinated solvent systems, supercritical fluid extraction, and aqueous two-phase extraction have been reported to enhance recovery yields and reduce polymer loss [2]. More recently, genetically engineered strains with weakened cell walls and autolytic systems have been explored as a means to simplify downstream processing and minimize extraction-related losses. These approaches collectively demonstrate promising pathways to improve both the efficiency and sustainability of PHA recovery.

## 4. Degradation of PHAs

**Figure 3:**
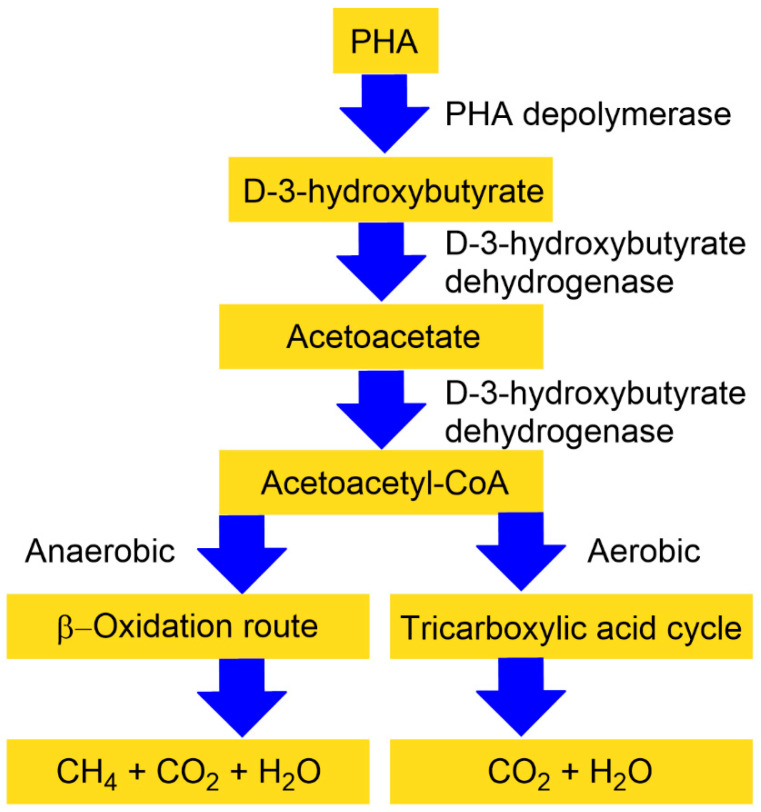
A simplified representation of extracellular PHA degradation under anaerobic and aerobic conditions. The tricarboxylic acid cycle is also often known as the Krebs cycle, or citric acid cycle (reprinted from reference [69], open access, Elsevier 2023).

One of the most significant benefits of PHAs compared to other bioplastics is their ability to completely biodegrade, even in marine environments. Research indicated that the biological breakdown of PHAs is 8–20 times faster than non-biological degradation [66]. A wide range of bacteria and fungi can degrade PHAs, and such organisms have been identified in varied ecosystems, including soil, compost, and aquatic environments [67]. Under aerobic conditions, such as in soil or seawater, PHAs decompose into carbon dioxide and water, whereas in anaerobic settings like sediments or landfills, they are converted into carbon dioxide and methane. The suggested degradation pathway is presented in Figure 3.

The process begins with microbial exo-enzymes that break down insoluble PHAs into smaller, water-soluble fragments such as oligomers. These oligomers, along with monomers, are then utilized by microorganisms as sources of carbon and energy, ultimately leading to complete mineralization [68]. This continuous cycle of PHA synthesis and degradation not only helps mitigate plastic pollution but also contributes to a circular economy by enabling materials to be repeatedly produced from renewable biological sources and returned to the environment through natural biodegradation without accumulating as waste [69].

## 5. Medical Application of PHA

PHAs are widely utilized across the medical sector, with applications ranging from preventive healthcare and diagnostic tools to surgical procedures, as evidenced by numerous patents [70]. They are incorporated into single-use medical items such as syringes and blood storage bags, and play a role in surgical materials, including sutures, tissue adhesives, and sealants. In orthopedics, PHAs are fashioned into devices like screws and fixation plates, while in prosthetics, they are employed for intraocular lenses, dental implants, breast implants, and cartilage repair systems. Their versatility extends further to artificial organs designed for either temporary or permanent support, such as synthetic kidneys and hearts. Additional uses include cardiovascular patches, nerve guidance conduits, PHB-coated (PHB: polyhydroxybutyrate) surgical meshes for hernia repair, and a variety of applications in reconstructive and skin-related surgeries.

### 5.1. Tissue Engineering

Tissue engineering has emerged as a multidisciplinary field that merges biomaterials with medical science to create functional tissue replacements. Its central aim is to regenerate or repair tissues by integrating living cells, bioactive compounds, and supportive biomaterials [71]. For biomaterials to be effective in tissue restoration, they must satisfy two essential criteria: (1) they should possess adequate mechanical strength to sustain organs during healing; and (2) they must provide a surface structure that encourages firm cell attachment and active proliferation. To achieve these requirements, researchers design scaffolds that mimic the architecture, spatial organization, and chemical environment of the natural extracellular matrix, thereby fostering cell growth and differentiation [72]. Tissues are broadly divided into two categories: hard tissue substitutes, such as bone and cartilage, and soft tissue substitutes, including vascular grafts and skin replacements [73]. The natural occurrence of 3-hydroxybutyrate (3HB) in the bloodstream, combined with the excellent biocompatibility of PHB, makes PHB particularly suitable for scaffold construction in tissue and organ repair [74]. Biopolymers like PHAs provide tunable mechanical properties, high compatibility with biological systems, minimal toxicity, and efficient biodegradability within the body. They have been utilized to fabricate scaffolds with improved mechanical resilience—such as screws, pins, sutures, and thin films—that supply nutrients to cells, stimulate tissue growth, and assist in repairing cartilage, skin, nerve pathways, and cardiovascular structures [75]. Furthermore, PHAs can be enhanced by blending with inorganic bioceramics or hydroxyapatite (HAp), producing advanced composite scaffolds and tissue products tailored for therapeutic and medical applications [76].

#### 5.1.1. Bone Tissue

Artificial prostheses, including bone grafts and substitutes, are vital in addressing osteochondral defects, congenital skeletal deformities, and in maintaining separation between articular surfaces and underlying bone layers. Bone tissue engineering, which emphasizes the regeneration of new bone, provides structural reinforcement while simultaneously stimulating cellular activity, thereby aiding in the correction of bone-related disorders. scl-PHAs, particularly PHB and PHBV (PHBV: poly(3-hydroxybutyrate-co-3-hydroxyvalerate), have been successfully applied to fabricate bone-supporting materials with strong mechanical properties [77]. To further enhance their performance, PHAs are frequently combined with inorganic or bioactive components—such as hydrogels, HAp, and other biocompatible polymers—resulting in improved tensile strength, elasticity, stress resistance, and compressive modulus, while also promoting osteoblast adhesion, proliferation, and differentiation in both laboratory and animal studies [78].

Additional bioactive reinforcements, including natural coral (NC) [79], sol–gel bioactive glass (SGBG), tricalcium phosphate (TCP) [80], and wollastonite (W) [81], have been incorporated into PHB and PHBV composites to further optimize bone regeneration. Among these, HAp is particularly significant, as it constitutes 65–70% of the natural bone matrix. In vitro experiments reveal that the mechanical strength, osteoblast activity, inflammatory response, and mineralization of PHB/HAp composites are strongly influenced by the proportion of HAp present [82]. Recent findings demonstrate that combining HAp with PHB produces bioresorbable porous scaffolds capable of enhancing both osteoconductivity and osteoinductivity [83]. In vivo studies further confirm that PHB reinforced with particulate HAp supports healthy bone remodeling without inducing chronic inflammation [84]. Moreover, PHB/HA scaffolds have been shown to maintain the viability and proliferation of human mesenchymal stromal cells (hMSCs) [85]. When PHB scaffolds seeded with hMSCs and coated with collagen-I or collagen-I/chondroitin sulfate were implanted in rats, vascularization occurred within the scaffold, and osteogenic markers such as osteonectin, osteopontin, and collagen I were expressed around PHB fibers—demonstrating effective bone growth and differentiation in vivo [86].

Blends of PHBV with hydroxyapatite have demonstrated significant promise in bone reconstruction at implant sites, where bone thickness increased from approximately 130 mm after one month to nearly 770 mm after six months [87]. When PHBV scaffolds were reinforced with hydroxyapatite nanoparticles, they exhibited notable improvements in compressive stiffness, mechanical strength, and in vitro bioactivity [88]. A novel scaffold design combining poly(lactic-co-glycolic acid) (PLGA) and PHBV—produced by embedding PHBV microspheres into a PLGA matrix through particle leaching—achieved high porosity, strong interconnectivity, enhanced compressive resistance, and promoted robust proliferation of human mesenchymal stem cells (hMSCs) [89]. Table 3 presents other examples of PHAs used for bone tissue engineering.

Hydrogels have also been investigated for scaffold construction because of their naturally porous architecture; however, their limited mechanical stability and weak bioactivity reduce their effectiveness. To overcome these shortcomings, researchers have developed tri-layered scaffolds that integrate PHB, hydroxyapatite, and protein-based hydrogels. These composite structures provide superior mechanical strength, enable effective cell encapsulation, and enhance the adaptability of bone cells to the scaffold environment [102] (see Table 4).

Electrospinning techniques have been employed to create hybrid scaffolds incorporating PHB, polycaprolactone (PCL, a biodegradable, semi-crystalline polyester widely used in biomaterials, tissue engineering, and drug-delivery systems), and bioactive glass, effectively merging the rigidity of PHB, the elasticity of PCL, and the biofunctional properties of 58S glass into a unified fibrous matrix (see Table 5) (58S bioactive glass is a sol–gel–derived bioactive glass made of about 60% silica (SiO_2_), 36% calcium oxide (CaO), and 4% phosphorus pentoxide (P_2_O_5_). It’s well known for bonding strongly with bone and stimulating biological activity. These engineered composites exhibited improved osteoblast survival, robust cellular interactions, and heightened alkaline phosphatase activity [77]. Extending beyond PHB and PHBV systems, scaffolds composed of poly(3-hydroxybutyrate-co-3-hydroxyhexanoate) (PHBHHx) have demonstrated notable compatibility with bone marrow cells. In comparison to PHB, PHBHHx scaffolds show superior adhesion to rabbit bone marrow cells, fostering cell proliferation and expressing osteogenic traits such as elevated alkaline phosphatase activity, calcium mineralization, collagen production, and rounded cellular morphology [115]. Furthermore, bone marrow stromal cells readily attach, proliferate, and undergo osteogenic differentiation when cultured on PHBHHx scaffolds in vitro, underscoring their strong biological affinity [116]. Incorporation of collagen into PHBHHx scaffolds has enabled the successful culture of hMSCs, reinforcing their potential utility in bone tissue engineering [117]. Despite hydroxyapatite’s recognized bioactivity and osteoconductivity, its combination with PHBHHx does not yield substantial improvements in mechanical strength or osteoblast performance [118]. A more advanced PHA variant, the terpolymer PHBVHHx, surpasses PHBV and PHBHHx in terms of flexibility, thermal resilience, and mechanical robustness. Its increased surface roughness and hydrophobicity enhance hMSC attachment more effectively than polylactic acid (PLA), PHBHHx, or tricalcium phosphate (TCP), leading to markedly higher cell proliferation rates [119].

Table 5 summarizes recent developments in the electrospinning-based fabrication of PHA and PHA composite nanofibers for tissue engineering applications. The listed studies highlight the growing diversity of PHA formulations—ranging from pure PHB to hybrid systems incorporating natural polymers, inorganic nanoparticles, and bioactive additives—and demonstrate how electrospinning enables the creation of highly porous, fibrous scaffolds that mimic the extracellular matrix. By comparing materials and publication years, the table illustrates current research trends, emerging composite strategies, and the expanding relevance of electrospun PHAs across various tissue engineering contexts.

To be effectively used in bone tissue engineering, polyhydroxyalkanoate-based composites must meet several essential criteria. First, they require sufficient mechanical strength and stiffness to withstand physiological loading and to provide temporary structural support during bone regeneration. Their biodegradation rate must be well-matched to the pace of new bone formation, ensuring gradual load transfer without premature collapse or prolonged persistence. PHA composites must also exhibit excellent biocompatibility, avoiding chronic inflammation while supporting osteoblast adhesion, proliferation, and differentiation. Incorporation of bioactive components—such as hydroxyapatite, tricalcium phosphate, or bioactive glass—should enhance osteoconductivity and osteoinductivity, promoting vascularization and mineralized tissue formation. Finally, the scaffold architecture must offer high porosity and interconnected pore networks to facilitate nutrient diffusion, cell infiltration, and neovascularization. Together, these criteria define the functional requirements for PHA-based materials to serve as reliable and effective platforms for bone repair and regeneration.

#### 5.1.2. Cartilage Tissue

Cartilage tissue engineering offers innovative approaches for the restoration of damaged articular cartilage. This specialized tissue, composed of chondrocytes, lines the ends of bones within synovial (diarthrodial) joints, where it functions to distribute mechanical stresses, facilitate smooth articulation, and maintain overall joint mobility. Because cartilage possesses the lowest cellular density of any human tissue and exhibits minimal intrinsic regenerative capacity, injury frequently results in impaired joint performance and progression toward osteoarthritis. Traditional surgical interventions—including microfracture, arthroscopic abrasion, and subchondral drilling—have been employed clinically but generally fail to reestablish native cartilage functionality [141,142]. To overcome these limitations, robust three-dimensional porous scaffolds fabricated from polymers such as PHB, PLA [143], polyglycolic acid (PGA) [144], PLGA [145], collagen [146], silk fibroin [147], chitosan [148], and their composites have been designed. These biomaterials provide a conducive framework for cellular proliferation and tissue integration, supporting both biomechanical stability and biochemical signaling [149].

A comprehensive review by Puppi et al. [150] highlighted the contributions of PHAs in cartilage regeneration. Porous scaffolds composed of PHBHHx/PHB blends have served as effective matrices for rabbit articular chondrocytes (RAC), demonstrating enhanced cell growth and proliferation compared to scaffolds of pure PHB. Blends containing 60% PHBHHx by weight exhibited superior mechanical performance relative to either polymer alone. These hybrid constructs not only improved chondrocyte viability and function but also facilitated the anchorage of type II collagen filaments and their penetration into deeper scaffold regions [151]. Complementary findings by Deng et al. [152] revealed that RAC cultured on PHB/PHBHHx blends expressed higher levels of collagen II mRNA compared to cells grown on neat PHB scaffolds. Moreover, extracellular collagen X—a marker associated with endochondral ossification—was progressively reduced as PHBHHx content increased, underscoring the role of blend composition in modulating chondrocyte activity (see Table 6).

#### 5.1.3. Cardiac Tissue

Cardiovascular disease continues to be one of the foremost causes of mortality and long-term disability across the globe [161]. To confront this pressing medical burden, cardiac tissue engineering has emerged as a pivotal research focus. Traditional surgical interventions—including defect reconstruction, revascularization, and patch closure—are widely practiced, yet the biomaterials employed must satisfy stringent criteria: they need to be mechanically robust, resistant to infection and degradation, biocompatible and non-toxic, sufficiently flexible, and available in diverse dimensions to support both vascular and cardiac repair procedures [162,163].

Among candidate materials, mcl-PHAs display distinctive attributes that render them highly suitable for cardiac applications. These polymers are elastic, exhibit elevated glass transition temperatures, integrate effectively with myocardial tissue, and possess the capacity to bind bioactive agents such as vascular endothelial growth factor, thereby promoting cellular adhesion, proliferation, and survival [164]. More recently, polymers including poly(4-hydroxybutyrate) (P4HB) and poly(3-hydroxyoctanoate) (P3HO) have been identified as promising options for addressing congenital heart anomalies, constructing vascular grafts, and fabricating heart valves. Additional bioplastics, such as poly(3-hydroxybutyrate-co-3-hydroxyvalerate-co-3-hydroxyhexanoate) (PHBVHHx) and poly(3-hydroxybutyrate-co-4-hydroxybutyrate) (PHB4HB), have also been utilized in the development of membranes and cardiac patches, respectively, underscoring their potential in cardiovascular tissue engineering [165] (see Table 7).

#### 5.1.4. Heart Valves

Cardiac tissue engineering provides a pathway for generating functional heart valve substitutes by molding biomaterials into valve-like architectures and subsequently seeding them with living cells [179]. Numerous biomaterials have been investigated for this application, including PHA, polylactic acid (PLA) [180], polycaprolactone (PCL) [181], polyglycolic acid (PGA) [182], and decellularized extracellular matrix, each offering a supportive microenvironment conducive to cellular proliferation and differentiation. Notably, mcl-PHAs exhibit superior flexibility compared to PLA and PGA, making them particularly advantageous for constructing leaflets in tri-leaflet valve designs [183]. The copolyester P(3HHx-co-3HO) (poly(3-hydroxyhexanoate-co-3-hydroxyoctanoate)), when paired with autologous cells in the pulmonary position, has yielded promising outcomes, characterized by only mild stenosis and the absence of thrombus formation [184]. Further advancements have been realized through blending PGA with P(3HHx-co-3HO) and reinforcing the composite with PHB, which improved the mechanical and biological properties of the scaffold, ultimately delivering enhanced performance [185].

#### 5.1.5. Vascular Grafts

Vascular grafting has been explored as a therapeutic strategy for managing diverse cardiovascular disorders. Yet, conventional synthetic graft materials frequently encounter limitations, including inadequate endothelialization, mismatched mechanical compliance, and rapid blockage under low-flow conditions, all of which contribute to postoperative complications [186]. In contrast, bioplastics such as PHAs present a compelling alternative, since their monomeric composition can be engineered to deliver optimal mechanical strength, biocompatibility, and predictable degradation behavior. Within this class, PHBHHx stands out for exhibiting enhanced physicochemical characteristics relative to PHB and PHBV (poly(3-hydroxybutyrate-co-3-hydroxyvalerate)) (see Table 8). Its suitability for blood-contact applications is underscored by diminished platelet adhesion, superior cytocompatibility and hemocompatibility, and reduced interactions with erythrocytes alongside lower hemolytic activity [187]. Even so, additional investigations—particularly those involving vascular graft implantation in animal models—remain essential to fully establish its translational potential in vascular tissue engineering and to resolve persistent challenges such as compliance mismatch [188].

#### 5.1.6. Artificial Blood Vessels

Due to their adjustable mechanical properties—including flexibility, elasticity, tensile strength, and the capacity to stimulate elastin synthesis—PHB and P3HB4HB (poly(3-hydroxybutyrate-co-4-hydroxybutyrate)) have been explored as promising biomaterials for constructing artificial blood vessels [200]. These polymers are regarded as strong contenders for vascular tissue applications. Moreover, advanced block copolymers with meticulously engineered architectures have been developed, notably alternating block polyurethanes synthesized from P3HB4HB-diol and poly(propylene glycol)-poly(ethylene glycol)-poly(propylene glycol), with 1,6-hexamethylene diisocyanate (HDI) serving as the capping agent [201].

#### 5.1.7. Skin Tissue Engineering

The search for advanced biomaterials capable of regenerating injured skin continues to pose a significant challenge in reconstructive medicine [202]. To address wound healing needs, both naturally derived polymers—such as collagen, alginic acid, hyaluronic acid, chitosan, and fucoidan—and synthetic alternatives, including Teflon, polyurethanes, and methyl methacrylate, have been utilized in the fabrication of artificial dressings [203]. Biopolymers are particularly advantageous because they emulate essential physiological roles of intact skin: they provide antimicrobial defense, enable gaseous exchange, sustain a moist healing environment, deliver mechanical reinforcement, exhibit sufficient tensile strength, and possess elasticity that allows them to adapt to irregular wound geometries [204]. Their application not only mitigates inflammation but also stimulates angiogenesis and expedites tissue repair. Within this context, PHAs, especially scl-PHAs, have been investigated for wound management [204]. Notably, PHA and PHB4HB have been employed in the design of wound dressings and surgical sutures, offering adequate mechanical resilience that makes them suitable for repairing muscle-fascial defects and cutaneous injuries [205] (see Table 9).

#### 5.1.8. Nerve Repair

Peripheral nerve damage frequently leads to partial or complete impairment of motor, sensory, and autonomic functions, and restoring these capabilities continues to pose a significant challenge for both clinicians and researchers. Clinical data indicate that only around 3% of patients recover sensory function, while fewer than 25% regain motor function after such injuries [219]. Although surgical repair remains the standard approach, lesions involving tissue gaps greater than 5 mm typically necessitate grafting. In this context, nerve guidance conduits (NGCs) have shown encouraging results, as they help prevent myofibroblast invasion, minimize scar formation, and provide a supportive framework for axonal regrowth [220]. Nevertheless, their clinical use is currently limited to relatively short nerve defects [221]. While several FDA-approved synthetic conduits exist, none are sanctioned for gaps exceeding 3 cm. The most employed natural biomaterials include collagen-based devices (NeuraGen^®^, Neuroflex^®^, NeuroMatrix^®^, RevolNerv^®^) and chitosan-derived conduits (Reaxon^®^), as highlighted in the review by Kornfeld et al. [222]. These biomaterial conduits generally facilitate functional recovery, though complications such as adverse effects and incomplete regeneration remain ongoing concerns.

PHAs have been widely explored in the context of nerve regeneration owing to their adaptable mechanical and physicochemical characteristics, which can be fine-tuned through fabrication techniques such as electrospinning, extrusion, and sheet rolling [223]. Among these, PHB has emerged as a particularly promising material in preclinical investigations, attributed to its biocompatibility, biodegradability, elasticity, and inherent piezoelectric behavior [224]. Importantly, PHB has also been shown to stimulate early vascularization following implantation [225]. Although its stability may restrict its utility in short-term applications, PHB remains highly suitable for long-term nerve repair, effectively bridging both small and extensive defects and offering a viable alternative to direct epineural suturing [226]. Evidence from rat sciatic nerve models demonstrates that PHB conduits can successfully restore 10 mm gaps within 30 days, characterized by minimal inflammatory response and robust axonal regrowth comparable to autologous grafts [227]. Complementary studies in rabbits, using the common peroneal nerve model, further validated PHB’s capacity to support regeneration across larger defects [227]. Moreover, the incorporation of trophic factors such as glial growth factor/neuregulin-1 (GGF/NRG1) significantly enhanced repair across 2–4 cm lesions, yielding favorable regenerative outcomes in both short- and long-term evaluations [228].

PHB conduits have been evaluated against fibrin glue conduits and further tested with internal fillers consisting of fibrin matrices seeded with Schwann cells (SCs) or mesenchymal stem cells, achieving successful repair of 10 mm nerve gaps in rat models [229]. In another approach, PHB strips populated with SCs were applied to bridge sciatic nerve defects, facilitating early axonal regeneration and enabling functional recovery within just two weeks [230]. Extended investigations lasting 12 months, in which PHB strips were enriched with either SCs or adipose-derived stem cells, revealed enhanced regenerative outcomes, including greater axonal penetration into distal stumps and reduced muscle atrophy compared to untreated controls [231]. Modifications of PHB through blending with chitosan minimized crystallization and allowed the fabrication of aligned fibers via electrospinning, thereby improving scaffold performance [232]. Additionally, composite structures combining PHB with synthetic polymers such as PCL have produced effective nerve guidance conduits [233].

Although PHB offers several advantages, it is hindered by drawbacks such as brittleness, pronounced crystallization, and limited hydrophilicity, all of which compromise its ductility and flexibility. To overcome these shortcomings, poly(3-hydroxybutyrate-co-3-hydroxyvalerate) (PHBV) was engineered through copolymerization of PHB with hydroxyvalerate, resulting in improved mechanical characteristics [234,235]. Compared to PHB, PHBV exhibits greater flexibility and is easier to process, though it remains constrained by a narrow processing window and a lower strain-at-break relative to petroleum-derived polymers [236]. In vivo studies using rat sciatic nerve models demonstrated that PHBV fibrous conduits successfully bridged 30 mm defects within four months, restoring skeletal muscle innervation and producing fiber diameters similar to those achieved with autografts [237,238]. The addition of SCs further enhanced regenerative outcomes [239,240]. Structurally, PHBV conduits are highly porous (~95%), biocompatible, and mechanically adaptable, capable of bending up to 180° and returning to their original form. Moreover, micropatterned PHBV wafers have facilitated both sensory and motor recovery in rats following 30 mm nerve gap repairs [241]. Beyond conduits, PHBV microspheres integrated with alginate hydrogel have supported the growth, differentiation, and maturation of neuroblastoma cells and fetal cortical neurons, underscoring their potential in neural tissue engineering [242].

Poly(3-hydroxybutyrate-co-3-hydroxyhexanoate) (PHBHHx), a notable PHA derivative, exhibits enhanced flexibility and mechanical resilience owing to the incorporation of hydroxyhexanoate (HHx) units within its polymer backbone. This material demonstrates excellent biocompatibility with neural stem cells and has been engineered into conduits featuring both uniform and non-uniform wall porosity, each successfully bridging 10 mm sciatic nerve gaps in rat models [243]. Although both architectures facilitated nutrient diffusion, conduits with non-uniform porosity offered superior mechanical integrity and more favorable biodegradation profiles. PHBHHx nanofiber scaffolds further revealed a strong affinity for neuronal stem and progenitor cells, effectively supporting their adhesion, viability, differentiation, and synapse formation [244]. Strikingly, when PHBHHx scaffolds seeded with neural stem cell/neural progenitor cell complexes were implanted into rats suffering from traumatic brain injury, they prevented glial scar development and instead promoted neurite outgrowth alongside measurable functional recovery [245]. Table 10 presents some other examples of the PHAs used for nerve regeneration.

### 5.2. Overview of Drug Delivery Systems and the Emergence of PHAs

In conventional drug administration, compounds disperse systemically rather than concentrating at the intended organ or tissue, which often diminishes therapeutic efficiency. To overcome these shortcomings, drug delivery systems (DDS) were introduced in the United States during the late 1960s. These polymer-based platforms were designed to maintain controlled release of drugs at optimal concentrations, direct therapeutic agents specifically to diseased sites while sparing healthy tissues, and enable externally triggered release mechanisms. They also simplified administration routes, most commonly through dermal or mucosal pathways. Collectively, DDS enhance solubility and bioavailability, mitigate toxicity, minimize adverse effects, and allow precise targeting of particular cells and tissues [253].

By the early 1990s, PHAs had gained recognition as promising materials for drug delivery applications, owing to their inherent biodegradability, biocompatibility, and favorable thermal processing properties. Their versatile chemical structures and modifiable side groups provide opportunities for tailoring functionality, making them suitable for a wide range of delivery devices. Because PHAs combine biocompatibility with biodegradability, they are well tolerated by human cells and tissues [254], which underpins their increasing adoption in drug delivery research and biomedical applications. Of all the PHAs, PHB and PHBV have been the most widely investigated for developing effective drug delivery platforms, including micro- and nanodevices, disks, implants, rods, and films [255].

#### 5.2.1. Antibiotic and Antimicrobial Applications

Microdevices such as microspheres and microcapsules have been developed to enable controlled drug delivery, including the administration of anesthetics, antibiotics, anti-inflammatory drugs, anticancer agents, hormones, steroids, and vaccines [256]. Biopolymeric systems such as P3HB4HB, PHBV, and PHB have been engineered into biodegradable, implantable rods and disks for localized antibiotic release in the treatment of chronic osteomyelitis, thereby reducing risks of post-surgical and implant-associated infections [257,258].

PHB and PHBV have been identified as promising candidates for drug delivery systems owing to their advantageous physicochemical properties, their neutrality toward platelet activity, and their compatibility with other polymers. Matrix-based carriers constructed from PHB and PHBV have been explored for the controlled release of numerous antibiotics and therapeutic compounds, including tetracycline, rifampicin, sulbactam-cefoperazone, gentamicin, sulperazone, rubomycin, and rhodamine B isothiocyanate [259]. Long-term investigations ranging from 15 to 60 days demonstrated that polymer molecular weight plays a critical role, with higher molecular weights correlating to slower drug release rates. Furthermore, the release kinetics and cumulative drug release are strongly influenced by the hydroxyvalerate (HV) content in PHBV copolymers [85]; increasing HV units accelerates drug release, while the amount of drug loaded directly modulates release behavior. Among the different PHA variants examined, P3HB4HB has exhibited the most favorable and controlled release characteristics [260].

#### 5.2.2. Antitumor Applications

Nanostructures derived from PHB and its copolymers have been developed as carriers for a wide range of molecular therapeutics, including anticancer drugs, hormones, and immunomodulators, all of which are capable of penetrating intracellular membranes [257]. PHB microspheres have been explored as carriers for anticancer therapeutics, with rubomycin-loaded formulations demonstrating effective inhibition of cell proliferation in Ehrlich’s carcinoma models [261].

PHBV has been especially prominent in drug delivery research. Docetaxel encapsulated within PHBV-based nanoparticles (NPs) exhibited potent cytotoxicity against MCF-7 breast cancer cells [262]. These nanoparticles, optimized through a Box–Behnken statistical design, displayed enhanced efficacy compared to free docetaxel, while offering improved safety features attributed to their negative surface charge, sustained-release kinetics, and passive tumor targeting via the enhanced permeability and retention (EPR) effect. Furthermore, when PHBV was blended with D-α-tocopheryl polyethylene glycol succinate (TPGS), the resulting docetaxel-loaded nanoparticles provided more controlled and prolonged release, thereby lowering required therapeutic doses and reducing systemic toxicity [263].

Recent progress in drug delivery has introduced targeting approaches, in which doxorubicin (DOX) encapsulated within PHBV nanoparticles is functionalized with cell-targeting peptides. This modification enhances nanoparticle localization in close proximity to cell nuclei, thereby producing stronger cytotoxic effects than either free DOX or non-targeted nanoparticle formulations. Importantly, PHA-based nanoparticles with diameters of approximately 200 nm exhibit intrinsic antiproliferative activity, a property that can be exploited in anticancer treatment strategies [264].

#### 5.2.3. Cardiovascular Applications

In cardiovascular applications, PHB blended with polyhydroxyoctanoate has been incorporated into drug-eluting stent coatings to mitigate arterial restenosis [265]. The intrinsic hydrophobicity and biodegradability of PHAs support the sustained release of hydrophobic cardiovascular drugs, making them suitable for long-term vascular therapies [266].

#### 5.2.4. Immunomodulatory Applications

Multilayered sandwich films composed of PHBHHx copolymers, loaded with thymopentin-phospholipid complexes, achieved controlled drug release lasting up to 42 days and produced enhanced immunomodulatory activity in rat models of immunosuppression [267].

#### 5.2.5. Neurological Applications

Early investigations have highlighted the promise of PHA-based nanoparticles for transporting therapeutic agents in the treatment of neurological disorders. PHBV nanoparticles have been explored for drug delivery in multiple sclerosis [268] and Parkinson’s disease [269], demonstrating their potential for crossing biological barriers and delivering neuroactive compounds.

#### 5.2.6. Manufacturing and Engineering Considerations

A variety of processing strategies are currently employed to fabricate PHA-derived microparticles and capsules for drug delivery applications [270]. Among these, the emulsion/solvent-evaporation method is widely adopted, as it provides enhanced control over drug encapsulation and stability [260]. Despite its utility, this technique suffers from notable drawbacks, particularly its inability to produce particles with a narrow and well-controlled size distribution, which can lead to unpredictable drug-release kinetics and reduced bioavailability [271]. Moreover, the formation of mesopores and micropores during fabrication, together with additional porosity generated through polymer degradation, often accelerates drug release and consequently limits the broader applicability of these delivery systems [36].

To overcome these limitations, innovative strategies have been introduced, including chemical modification, copolymer development, and surface functionalization with targeting ligands, all of which improve encapsulation efficiency, enhance chemical stability, and prolong release profiles [272]. Advances in automated manufacturing have further enabled the production of PHA-based particles with highly uniform sizes and reproducible characteristics, which is essential for ensuring consistent performance in downstream applications such as drug delivery, cell encapsulation, and controlled release studies [273]. Additionally, the conjugation of bioactive molecules or targeting moieties to particle surfaces has been shown to modulate cellular uptake, making these systems particularly valuable in the design of next-generation cancer therapeutics [274].

#### 5.2.7. Material Limitations and Strategies for Improvement

The biodegradability of PHB and PHBV allows them to facilitate controlled drug release via surface erosion. Nonetheless, their relatively high melting points and crystalline nature restrict drug-loading capacity and reduce release efficiency [275]. To overcome these limitations, strategies such as blending PHB with other polymers or creating copolymers have been employed. These modifications yield softer and more resilient materials with reduced melting points, thereby enhancing their practicality and effectiveness in drug delivery applications.

#### 5.2.8. PHB Copolymers

An amorphous copolymer composed of poly(3-hydroxybutyrate)-poly(ethylene glycol)-poly(3-hydroxybutyrate) (PHB-PEG-PHB) has been engineered to function as a delivery platform for hydrophobic therapeutic agents [276]. Relative to pure PHB, this copolymer exhibits enhanced drug-loading efficiency while retaining comparable biodegradability and controlled release behavior. The incorporation of PEG not only prolongs systemic circulation time [277] but has also been applied in the design of copolymer-based formulations for peptide-loaded nanoparticles [278]. Remarkably, in vivo experiments employing PHB-PEG nanoparticles encapsulating the anticancer peptide NuBCP-9 achieved a 90% reduction in tumor size within an Ehrlich syngeneic mouse model.

A biodegradable cationic amphiphilic copolymer, PHB-b-poly(2-(dimethylamino)ethyl methacrylate) (PHB-b-PDMAEMA), has been utilized for the simultaneous delivery of paclitaxel and a Bcl-2 converting gene, successfully overcoming mechanisms of drug resistance [279]. These dual-delivery constructs achieved notable transfection efficiency and significantly increased apoptosis in drug-resistant HepG2 cancer cells. In parallel, PHB-b-PDMAEMA systems were also shown to suppress resistance pathways mediated by both p-glycoprotein and Bcl-2 proteins [280]. An ideal anticancer therapy selectively eliminates malignant cells while sparing healthy ones, and PHB/PEG-Nickel Oxide (NiO) nanocomposites encapsulating norfloxacin have demonstrated this selective cytotoxicity—exerting potent effects against cancer cells while leaving normal cells unaffected [281].

To further minimize adverse impacts on healthy tissues, injectable thermogelling PHB-based copolymers have been engineered. The PEG-PPG-PHB triblock polymer exhibits thermogelling properties, strong biocompatibility, and enables sustained release of chemotherapeutics such as paclitaxel and doxorubicin. Direct intratumoral administration of this PTX-thermogel produced greater tumor regression compared to either free drug or thermogel alone [282].

PHBV copolymers have also been explored for advanced drug delivery. An mPEG-PHBV copolymer synthesized via transesterification generated biocompatible nanoparticles capable of encapsulating and gradually releasing hydrophobic drugs. Furthermore, a PHBV-hyperbranched polyethylenimine (PEI) copolymer has been evaluated as a non-viral vector for siRNA delivery. This platform achieved superior transfection efficiency and reduced cytotoxicity compared to branched-PEI across two cell lines, while demonstrating in vitro luciferase gene silencing on par with Lipofectamine 2000 [283].

#### 5.2.9. PHA Blends

Nanoparticles fabricated from poly(3-hydroxybutyrate-co-3-hydroxyvalerate)/poly(lactic-co-glycolic acid) (PHBV/PLGA) blends and loaded with the recombinant protein teriparatide have been investigated as a therapeutic strategy for osteoporosis [284]. The optimized formulation exhibited a prolonged in vitro release profile, with the encapsulated teriparatide maintaining stability throughout the duration of release. An alternative design involved injectable gelatin-based hydrogels incorporating PHB nanoparticles. In this dual drug-delivery system, naproxen sodium was encapsulated within PHB nanoparticles, while curcumin was integrated into the hydrogel matrix. In vitro evaluations confirmed that this configuration achieved sustained release of both therapeutic agents [285]. Similarly, injectable hydrogels containing PHBV nanoparticles have been developed. In this case, nafarelin-loaded nanoparticles were dispersed within a sodium alginate/Poloxamer 407 solution, and in vitro testing demonstrated continuous release of nafarelin for as long as 60 days [286].

#### 5.2.10. Fiber Membranes

Electrospun PHB-PEO fiber membranes incorporating different concentrations of chlorhexidine (CHX) (1%, 5%, and 10%) demonstrated antibacterial efficacy against *E. coli* and *S. aureus*. Interestingly, the 1% CHX formulation achieved both a sustained release profile and a faster initial release compared to higher loadings [287]. In a similar vein, solvent-free melt electrospinning was used to fabricate PLLA-PHB (PLLA: poly(L-lactic acid)) fibers containing dipyridamole. These fibers exhibited irregular surface textures and variable diameters, while the inclusion of PHB reduced PLLA crystallinity. In vitro release experiments revealed that drug release was predominantly controlled by diffusion through the polymer matrix, although ester bond hydrolysis also contributed as the polymer degraded [288].

Moving beyond conventional DDS, PHAs—particularly mcl-PHAs—have been explored for transdermal drug delivery systems (TDDS) (see Table 11). Such systems encompass drug encapsulation, cellular uptake via endocytosis, retention within tissues, controlled release, and activation in vivo [289]. Their performance in these applications is closely tied to physicochemical parameters such as degradation rate, solubility in water, molecular weight, and their capacity to be chemically grafted with other biodegradable polymers like PCL, PLA, and PLGA. A notable illustration is PEG-PCL micelles loaded with gold nanoparticles, which, upon stimulation with near-infrared radiation (NIR), successfully induced apoptosis in cancer cells during in vitro studies [290].

**Figure 4:**
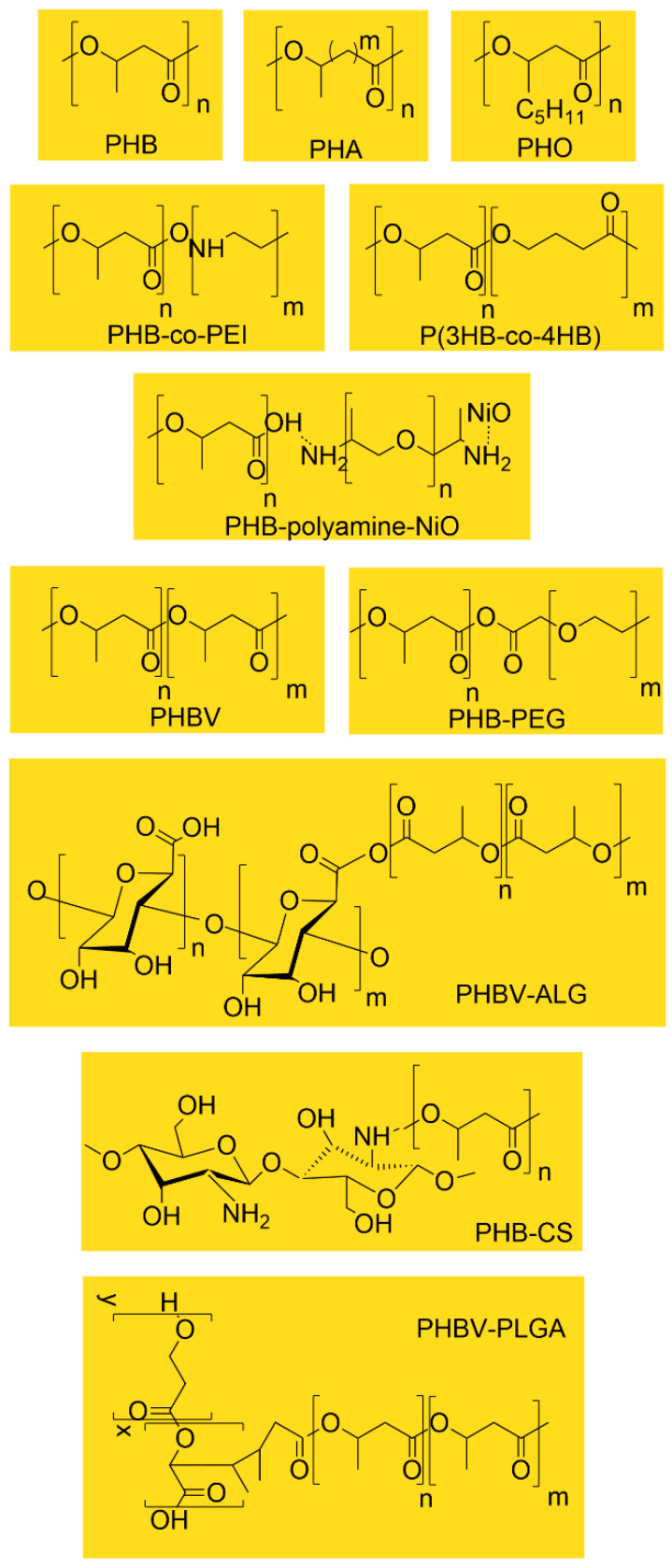
Representative chemical structures of PHAs and PHA-based copolymers used in drug delivery systems.

To facilitate comparison among the various PHA-based systems used in drug delivery, Figure 4 summarizes the chemical structures of the principal homopolymers and copolymers discussed in this section. Differences in side-chain length, co-monomer composition, and functional modifications (e.g., PEGylation, PEI grafting, metal-oxide conjugation) directly influence hydrophobicity, crystallinity, degradation rate, and drug-loading capacity. These structural distinctions underpin the diverse performance profiles observed across PHA-based nanoparticle and composite formulations.

## 6. Clinical Trials and Regulatory Approval of Pha-Based Devices

Regulatory approval has already been secured for P(4HB), a scl-PHAs commercialized under the name TephaFLEX^®^, in both the United States and Europe [302]. In the U.S., the Food and Drug Administration (FDA) authorized TephaFLEX^®^ for clinical use in surgical sutures as early as 2007 [64]. Another clinically available derivative of P(4HB) is the PHASIX™ plug and patch, specifically developed for the treatment of inguinal hernias [77]. Looking ahead, it is expected that ongoing and future clinical trials will further investigate the therapeutic potential of PHA-derived prototypes [303].

## 7. Challenges

PHAs have gained significant attention due to their biocompatibility, biodegradability, and versatility, positioning them as promising materials for both large-scale industrial production and advanced medical applications. Their key physicochemical properties—such as glass transition temperature, melting point, tensile strength, elongation capacity, and elastic modulus—are strongly shaped by monomer composition and molecular weight, making careful optimization essential for achieving targeted performance. High molecular weight copolymers, in particular, offer improved mechanical and thermal characteristics, though their use in biomedical settings still requires caution, as certain formulations or impurities may trigger immune responses and therefore demand thorough preclinical evaluation. At the same time, the broader adoption of PHAs in clinical practice continues to be limited by challenges in cost-effective scale-up and consistent production of medical-grade materials. Microbial fermentation and downstream purification remain resource-intensive, with high energy demands and solvent use contributing to elevated costs. Strategies to address these barriers include altering feedstock composition, utilizing low-cost agricultural residues or wastewater organics, refining culture conditions, engineering robust microbial strains, adopting continuous fermentation systems, and developing solvent-free or enzymatic recovery methods. Collaborative efforts across academia, industry, and government, supported by biorefinery integration and public funding, will be essential to make PHA production economically viable. Regulatory approval adds another layer of complexity, as variations in molecular weight, crystallinity, and degradation rates complicate standardization, and current frameworks often require case-by-case evaluations. Long-term biodegradation studies, GMP-compliant manufacturing, and harmonized testing protocols are needed to streamline approval pathways and build clinical trust. Looking ahead, innovations such as smart PHAs responsive to stimuli, hybrid composites with ceramics or nanomaterials, and 3D-printed patient-specific implants are reshaping their role in healthcare. Beyond clinical performance, PHAs also support sustainability by enabling a circular bioeconomy—produced from renewable feedstocks, degrading into non-toxic compounds, and reducing environmental burdens through eco-friendly medical devices. The journey of PHAs from microbial byproducts to multifunctional biomaterials underscores both the technical and regulatory challenges ahead, but continued investment and interdisciplinary collaboration are paving the way for PHAs to evolve into the gold standard of sustainable medical and industrial polymers.

## 8. Conclusions

PHAs have emerged as one of the most versatile and promising biomaterials in modern medicine. Their rare combination of biocompatibility, biodegradability, mechanical adaptability, and functional tunability positions them as ideal candidates for applications ranging from drug delivery systems and wound dressings to orthopedic implants and tissue engineering scaffolds. PHAs can be fabricated into diverse formats—nanoparticles, microspheres, hydrogels, and films—each offering unique advantages for sustained release, targeted therapy, and regenerative support. Their ability to degrade into non-toxic metabolites, promote cellular activities, and align with therapeutic timelines underscores their clinical relevance, while their integration into sutures, stents, and fixation devices highlights their structural reliability and biological harmony.

The rise in PHAs reflects the power of interdisciplinary collaboration. Advances in biotechnology have enabled scalable microbial production using engineered strains and waste-derived feedstocks. Materials science has refined its mechanical properties, degradation profiles, and bioactivity through blending, copolymerization, and surface modification. Medicine has validated their efficacy in wound healing, tissue regeneration, and implant performance, ensuring that PHA-based devices are both biologically effective and clinically viable. This convergence of disciplines has created a feedback loop of innovation, where biological insights inform material design and material capabilities expand therapeutic possibilities.

At their core, PHAs represent a paradigm shift in how we approach biomaterials—transforming microbial byproducts and waste carbon sources into high-value medical solutions. They embody the principles of circular bioeconomy and regenerative design, offering a model of responsible innovation that bridges clinical performance with ecological stewardship. In a world facing plastic pollution, resource scarcity, and healthcare inequities, PHAs demonstrate that high-performance materials can also be sustainable, safe, and socially responsible.

Looking ahead, PHAs may serve as the foundation for next-generation smart biomaterials—responsive, personalized, and ecologically attuned. Their potential integration with biosensors, targeted therapies, and regenerative platforms places them at the forefront of future medicine. More than just materials, PHAs embody a philosophy of innovation rooted in harmony between biology and technology, resilience in design, and renewal of both human health and planetary well-being.

Realizing their full potential will require continued investment in research, infrastructure, and policy, alongside rethinking production paradigms and regulatory frameworks. The rewards—safer therapies, cleaner environments, and more equitable healthcare—are well worth the effort. Ultimately, PHAs offer a vision of medicine that heals not only the body but also the planet, standing as a powerful example of what is possible when science, sustainability, and humanity converge.

Unlike previous reviews that concentrate mainly on drug-delivery technologies, this review positions PHAs within a wider biomedical and ecological framework. By linking material origin, biodegradability, clinical readiness, and environmental responsibility, we highlight PHAs as a platform that unites sustainable manufacturing with therapeutic function. This integrated perspective underscores their potential not only as drug carriers but as versatile, next-generation biomaterials capable of supporting repair, regeneration, and patient-centered healthcare.

## Figures and Tables

**Table 1:** Applications, useful life, and degradation time of different petrochemical plastics are compared to polyhydroxyalkanoates (PHAs). The degradation time varies depending on the shape and thickness of the plastic object, its application, and the environment in which it is located (e.g., land, sea, temperature, etc.) (Reprinted from reference [1], MDPI 2024).

Plastic	Applications	Usage Time	Degradation Time
PET (Polyethylene terephthalate)	Bottles and other plastic containers	1–3 years	500–1000 years
HDPE (High-density polyethylene)	Pipelines, bottles	5–35 years	250–5000 years
LDPE (Low-density polyethylene)	Plastic wrappers and bags	1–3 years	150 years
PVC (Polyvinyl chloride)	Pipelines and other uses in construction	35 years	>1000 years
PP (Polypropylene)	Textiles, packaging, automotive components	5–15 years	50–800 years
PHAs (Polyhydroxyalkanoates)	Bags, packaging, medical implants	-	<1 year

**Table 2:** Properties and applications of different PHAs based on the size of their side chain (R) (Reprinted from reference [1], MDPI 2024).

Classification	Properties	Applications	Examples	References
Short-chain PHAs (4–5 carbons)	Fragile High melting point Greater biodegradability Biocompatibility Rigidity High crystallinity	Biofuel production Tissue engineering Drugs encapsulation	Poly(3-hydroxybutyrate) Poly(3-hydroxyvalyrate)	[28,29,30]
Medium-chain PHAs (6–14 carbons)	More elastic Low melting point Biodegradability Biocompatibility Semi-crystalline or amorphous	Fertilizer encapsulation Adhesives Coatings Soft tissue engineering	Poly(3-hydroxyoctanoate) Poly(3-hydroxyexanoate)	[31,32,33,34]
Long-chain PHAs > 14 carbons)	Elastic Low melting point Low glass transition temperature Low crystallinity Low tensile strength	Packaging materials	Poly(3-hydroxyhexadecanoate)	[35]

**Table 3:** Some PHAs used for bone tissue engineering applications.

Biopolymer *	Application	Year of Publication	References
PHA/β-TCP	In vitro on dental pulp stem cells and osteoblast-like cells	2022	[90]
PLA-20PHA/10nHAp	Medical device in bone tissue engineering	2023	[91]
P(3HB); P(3HO-co-3HD-co-3HDD) + HAp	Bone tissue	2021	[92]
PHB/PHA/PLA/HAp	Biocompatible scaffold	2024	[93]
PCL/PHBV; PCL/PHBV/HAp	Scaffold for bone regeneration	2021	[94]
PHB-K/nHAp	Scaffold for bone regeneration	2022	[95]
PLA/PHA/HAp	Bioactive implant in bone regeneration	2025	[96]
PHB/Hap/ALG/MSC	Scaffold supports MSC growth and osteogenic differentiation	2020	[97]
PHBV/HAp	Scaffold for bone tissue regeneration	2021	[98]
PHB/HAp	Scaffold for bone regeneration applications	2016	[99]
PHB/HAp	Scaffold for bone formation	2017	[100]
PHBV/CTS/HAp8	Scaffold for bone replacement therapies	2015	[101]
PHA/HAp	Scaffold for MC3T3-E1 cells differentiation	2019	[77]

* Abbreviations: PHA/β-TCP refers to polyhydroxyalkanoate with β-tricalcium phosphate; PLA-20PHA/10nHAp denotes polylactic acid containing 20% polyhydroxyalkanoate and 10% nano-hydroxyapatite; P(3HB) indicates poly(3-hydroxybutyrate), while P(3HO-co-3HD-co-3HDD) represents poly(3-hydroxyoctanoate-co-3-hydroxydecanoate-co-3-hydroxydodecanoate) combined with hydroxyapatite (HAp). PHB/PHA/PLA/HAp corresponds to poly(3-hydroxybutyrate), polyhydroxyalkanoate, polylactic acid, and hydroxyapatite; PCL/PHBV and PCL/PHBV/HAp refer to polycaprolactone with poly(3-hydroxybutyrate-co-3-hydroxyvalerate), with or without hydroxyapatite. PHB-K/nHAp denotes potassium-modified poly(3-hydroxybutyrate) with nano-hydroxyapatite; PLA/PHA/HAp represents polylactic acid, polyhydroxyalkanoate, and hydroxyapatite. PHB/HAp/ALG/MSC indicates poly(3-hydroxybutyrate), hydroxyapatite, alginate, and mesenchymal stem cells; PHBV/HAp and PHB/HAp refer to poly(3-hydroxybutyrate-co-3-hydroxyvalerate) or poly(3-hydroxybutyrate) with hydroxyapatite. PHBV/CTS/HAp denotes poly(3-hydroxybutyrate-co-3-hydroxyvalerate) with chitosan and hydroxyapatite, while PHA/HAp refers to polyhydroxyalkanoate combined with hydroxyapatite.

**Table 4:** Some PHA–hydrogel hybrids used for tissue engineering applications.

Biopolymer *	Application	Year of Publication	References
PHBV/PNFs/CMCht-SF:PEDGE	Cartilage tissue regeneration	2020	[103]
PHBV/SA; PHBV/CA	Skin biomedical applications	2022	[104]
Chitosan/loofah/PHBV	Scaffolds for engineering meniscus tissue	2022	[105]
PHBV	Scaffolds for drug release and tissue regeneration	2025	[106]
PHBV + P34HB	Wound healing and tissue implant	2025	[107]
PHB-Cs/rGO	Bone tissue regeneration	2025	[108]
PHB/CHIT	Treatment of chondral and osteochondral defects	2021	[109]
PHB-PEG-NFC	Material for prosthetic devices	2023	[110]
PHB/BC	Scaffold for new bone formation in calvarial defects	2025	[111]
PHA/PHU10U	Tissue engineering scaffold	2019	[112]
Chitin/PHBV	Scaffold for skin tissue regeneration	2012	[113]
PHB/HAp + protein hydrogels	In vivo bone regeneration	2016	[101]
CS/PHBV/CP	Scaffold for adhesion and chondrogenic differentiation	2015	[114]

* Abbreviations: PHBV/PNFs/CMCht-SF:PEDGE refers to poly(3-hydroxybutyrate-co-3-hydroxyvalerate) combined with plant nanofibers and carboxymethyl chitosan–silk fibroin crosslinked with polyethylene diglycidyl ether; PHBV/SA and PHBV/CA denote poly(3-hydroxybutyrate-co-3-hydroxyvalerate) with sodium alginate or calcium alginate, respectively. Chitosan/loofah/PHBV represents a composite of chitosan, loofah-derived fibers, and poly(3-hydroxybutyrate-co-3-hydroxyvalerate). PHBV alone refers to poly(3-hydroxybutyrate-co-3-hydroxyvalerate), while PHBV + P34HB indicates a blend of PHBV with poly(3-hydroxybutyrate-co-4-hydroxybutyrate). PHB-Cs/rGO denotes poly(3-hydroxybutyrate) with chitosan and reduced graphene oxide; PHB/CHIT refers to poly(3-hydroxybutyrate) with chitin. PHB-PEG-NFC represents poly(3-hydroxybutyrate) combined with polyethylene glycol and nanofibrillated cellulose, and PHB/BC denotes poly(3-hydroxybutyrate) with bacterial cellulose. PHA/PHU10U refers to polyhydroxyalkanoate blended with a polyurethane-based component (PHU10U). Chitin/PHBV indicates a composite of chitin and poly(3-hydroxybutyrate-co-3-hydroxyvalerate). PHB/HAp + protein hydrogels describe poly(3-hydroxybutyrate) with hydroxyapatite and protein-based hydrogels. CS/PHBV/CP denotes chitosan, poly(3-hydroxybutyrate-co-3-hydroxyvalerate), and calcium phosphate.

**Table 5:** Electrospinning-based synthesis of PHAs for tissue engineering applications.

Biopolymer *	Application	Year of Publication	References
PHB	Scaffolds for tissue engineering	2025	[120]
PHB/gelatin	Scaffolds for bone regeneration	2024	[121]
Chitosan/PHB	Membranes with antibacterial activity	2024	[122]
PHB-starch-MWCNTs	Scaffolds for bone tissue engineering	2022	[123]
PHB-lignin/cellulose	Scaffolds for bone tissue engineering	2023	[124]
PHB-zein	Scaffolds for bone tissue engineering	2023	[125]
PHB/starch	Scaffolds for bone tissue engineering	2021	[126]
PLA/PHA/P(3HO)	Scaffolds for tissue engineering	2023	[127]
PHBV with milk and molasses organic residues	Scaffolds for tissue engineering and drug delivery	2025	[128]
PHBV	Scaffold for tissue engineering and wound dressing	2023	[129]
PHB/Fe_3_O_4_-rGO	Scaffolds for bone tissue engineering	2022	[130]
PHB	Treatment system for diabetes mellitus	2021	[131]
HAp/PHBV/gelatin	Matrices for bone tissue engineering	2024	[132]
PHB-Nb_2_O_5_	Membranes for bone tissue engineering	2025	[133]
P4HB	Microspheres for bone tissue regeneration	2021	[134]
PHBV-PEG-melatonin	Scaffolds for bone tumor treatment	2023	[135]
BaTiO_3_/PHB	Scaffolds for bone tissue engineering	2024	[136]
LiBH_4_-PHBV	Scaffolds for tissue engineering	2022	[137]
PC/PHBV	Scaffolds for cartilage tissue engineering	2022	[138]
PLLA/PHB	Scaffolds for bone tissue engineering	2021	[139]
Lignin-PHB	Nanofibers with antioxidant activity	2019	[140]

* Abbreviations: PHB refers to poly(3-hydroxybutyrate), while PHB/gelatin denotes a composite of poly(3-hydroxybutyrate) and gelatin. Chitosan/PHB represents a blend of chitosan with poly(3-hydroxybutyrate). PHB-starch-MWCNTs indicates poly(3-hydroxybutyrate) combined with starch and multi-walled carbon nanotubes, whereas PHB-lignin/cellulose refers to poly(3-hydroxybutyrate) with lignin and cellulose fibers. PHB-zein denotes poly(3-hydroxybutyrate) blended with the plant protein zein, and PHB/starch represents a poly(3-hydroxybutyrate)–starch composite. PLA/PHA/P(3HO) corresponds to polylactic acid, polyhydroxyalkanoate, and poly(3-hydroxyoctanoate). PHBV with milk and molasses organic residues refers to poly(3-hydroxybutyrate-co-3-hydroxyvalerate) produced using dairy and molasses-derived substrates. PHBV alone denotes poly(3-hydroxybutyrate-co-3-hydroxyvalerate). PHB/Fe_3_O_4_-rGO indicates poly(3-hydroxybutyrate) combined with magnetite (Fe_3_O_4_) and reduced graphene oxide. HAp/PHBV/gelatin represents hydroxyapatite, poly(3-hydroxybutyrate-co-3-hydroxyvalerate), and gelatin. PHB-Nb_2_O_5_ denotes poly(3-hydroxybutyrate) reinforced with niobium pentoxide nanoparticles. P4HB refers to poly(4-hydroxybutyrate). PHBV-PEG-melatonin indicates poly(3-hydroxybutyrate-co-3-hydroxyvalerate) combined with polyethylene glycol and melatonin. BaTiO_3_/PHB denotes barium titanate with poly(3-hydroxybutyrate). LiBH_4_-PHBV refers to lithium borohydride incorporated into poly(3-hydroxybutyrate-co-3-hydroxyvalerate). PC/PHBV represents polycarbonate blended with PHBV, while PLLA/PHB denotes poly(L-lactic acid) combined with poly(3-hydroxybutyrate). Lignin-PHB refers to a composite of lignin and poly(3-hydroxybutyrate).

**Table 6:** Cartilage tissue engineering applications for PHAs.

Biopolymer *	Application	Year of Publication	References
PHBV/PNFs/CMCht-SF	Scaffold for chondrogenic differentiation	2020	[102]
PHBV-g-QUE	Scaffold for clinical treatment of cartilage defects	2021	[153]
Genipin-PHBV/loofah/CS	Scaffolds for osteochondral tissue engineering applications	2023	[154]
PHBV-Bioglass	Improves the characteristics of CPC-based tissue-engineered cartilages in vivo (CPC: calcium phosphate cement)	2022	[155]
PHBV-g-Rg1	Scaffold applied in cartilage tissue engineering	2020	[156]
PHBV-TGF-β3	System for cartilage regenerative therapy	2025	[157]
PHA-WJ-MSCs	System for cartilage repair therapy	2025	[158]
MWNTs-PHB-chitosan	Scaffold for adhesion and chondrocyte proliferation	2020	[159]
PHBV-BaTiO_3_	Scaffolds for cartilage regeneration	2019	[160]

* Abbreviations: PHBV/PNFs/CMCht-SF refers to poly(3-hydroxybutyrate-co-3-hydroxyvalerate) combined with plant nanofibers and carboxymethyl chitosan–silk fibroin. PHBV-g-QUE denotes PHBV grafted with quercetin. Genipin-PHBV/loofah/CS represents genipin-crosslinked PHBV combined with loofah-derived fibers and chitosan. PHBV-Bioglass refers to poly(3-hydroxybutyrate-co-3-hydroxyvalerate) reinforced with bioactive glass. PHBV-g-Rg1 indicates PHBV grafted with ginsenoside Rg1, while PHBV-TGF-β3 denotes PHBV functionalized with transforming growth factor-β3. PHA-WJ-MSCs refers to polyhydroxyalkanoate scaffolds seeded with Wharton’s jelly–derived mesenchymal stem cells. MWNTs-PHB-chitosan describes a composite of multi-walled carbon nanotubes, poly(3-hydroxybutyrate), and chitosan. PHBV-BaTiO_3_ denotes poly(3-hydroxybutyrate-co-3-hydroxyvalerate) reinforced with barium titanate.

**Table 7:** Cardiac tissue engineering applications for PHAs.

Biopolymer *	Application	Year of Publication	References
PHA	Printed structure and potential application in medical devices	2023	[166]
PHBV/PCL	Patches in arterial implantation models of arterial reconstruction	2020	[167]
PHBV/PCL(VEGF-bFGF-SDF)^Hep/llo^	Implanted tissue-engineered carotid artery graft into sheep	2021	[168]
MCL-PHAs with hPSC-CMVECs	The system can support cardiac tissue regeneration and functional recovery	2025	[169]
MCL-PHAs with hPSC-CMs	Cardiac patches that matured hPSC-CMs and promoted vascular regeneration	2024	[170]
PLA/PHO	Fibrous biomaterial scaffold for tissue engineering application	2023	[126]
P(3OH)	System for cardiac tissue engineering	2018	[171]
MCL-PHAs	Delivery for CPCs and regenerative myocardial infarction (CPC: calcium phosphate cement)	2018	[172]
MCL-PHAs	Scaffold fabrication and application of MCL-PHAs in cardiac patches for myocardial repair	2017	[173]
PHB	Scaffold for cardiac tissue engineering	2014	[174]
P(3HB-co-4HB)	Scaffold for tissue engineering	2022	[175]
P(3HB-co-4HB)	A peptide-functionalized scaffold that can influence cardiac-related cell behavior	2020	[176]
PHA	Biomaterial for heart valve scaffold	2004	[177]
PHBHHx	Hybrid valve system for valve replacement	2007	[178]

* Abbreviations: PHA refers to polyhydroxyalkanoate. PHBV/PCL denotes a composite of poly(3-hydroxybutyrate-co-3-hydroxyvalerate) and polycaprolactone. PHBV/PCL(VEGF-bFGF-SDF)Hep/llo represents PHBV/PCL scaffolds loaded with vascular endothelial growth factor, basic fibroblast growth factor, stromal-derived factor, heparin, and iloprost. MCL-PHAs with hPSC-CMVECs refers to medium-chain-length polyhydroxyalkanoates combined with human pluripotent stem cell–derived cardiac microvascular endothelial cells, while MCL-PHAs with hPSC-CMs denotes the same polymer type combined with human pluripotent stem cell–derived cardiomyocytes. PLA/PHO indicates a blend of polylactic acid and polyhydroxyoctanoate. P(3OH) refers to poly(3-hydroxyoctanoate). PHB denotes poly(3-hydroxybutyrate). P(3HB-co-4HB) represents poly(3-hydroxybutyrate-co-4-hydroxybutyrate). PHBHHx refers to poly(3-hydroxybutyrate-co-3-hydroxyhexanoate).

**Table 8:** Vascular tissue engineering applications for PHAs.

Biopolymer *	Application	Year of Publication	References
PHBV/PCL-GFmix	Formation of a biodegradable vascular graft in the carotid artery of sheep	2020	[189]
PHBV/PCL	Implanted vascular grafts with regenerative potential	2023	[190]
PHB/HA-MA	Scaffold design for vascular tissue engineering	2023	[191]
ePTFE/PHB	Self-powered and sensing vascular grafts	2023	[192]
PHBV/PCL/VEGF	Vascular grafts implanted in vivo with VEFG, which improves mechanical performance and endothelialization (VEFG: vascular endothelial growth factor)	2016	[193]
PHBV + P34HB	Scaffold with enzymatically tunable degradability rates	2024	[194]
3-HB	Oral administration that attenuates atherosclerosis in mice	2021	[195]
PHO/BC	Tissue-engineered blood vessels	2017	[196]
PHBV/PLC	Implantation into the rad abdominal aorta, used as a vascular graft	2015	[197]
PHBHHx	Vascular grafts as small-diameter vascular grafts	2017	[198]
P3HB4HB	Scaffolds for artificial blood vessels	2008	[195]
PHO/a-PHB; PHB/a-PHB	Polymeric material for cardiovascular engineering	2012	[199]

* Abbreviations: PHBV/PCL-GFmix refers to poly(3-hydroxybutyrate-co-3-hydroxyvalerate) and polycaprolactone loaded with a mixed growth-factor formulation. PHBV/PCL denotes a composite of poly(3-hydroxybutyrate-co-3-hydroxyvalerate) and polycaprolactone. PHB/HA-MA represents poly(3-hydroxybutyrate) combined with hydroxyapatite modified with methacrylate groups. ePTFE/PHB refers to expanded polytetrafluoroethylene combined with poly(3-hydroxybutyrate). PHBV/PCL/VEGF denotes PHBV/PCL scaffolds incorporating vascular endothelial growth factor. PHBV+P34HB indicates a blend of poly(3-hydroxybutyrate-co-3-hydroxyvalerate) with poly(3-hydroxybutyrate-co-4-hydroxybutyrate). 3-HB refers to 3-hydroxybutyrate. PHO/BC represents polyhydroxyoctanoate combined with bacterial cellulose. PHBV/PLC denotes poly(3-hydroxybutyrate-co-3-hydroxyvalerate) blended with poly(L-caprolactone). PHBHHx refers to poly(3-hydroxybutyrate-co-3-hydroxyhexanoate). P3HB4HB denotes poly(3-hydroxybutyrate-co-4-hydroxybutyrate). PHO/a-PHB and PHB/a-PHB refer to polyhydroxyoctanoate or poly(3-hydroxybutyrate) combined with amorphous poly(3-hydroxybutyrate).

**Table 9:** Wound healing tissue engineering using PHAs.

Biopolymer *	Application	Year of Publication	References
PHB/Collagen	Treatment of burn wounds	2025	[206]
PHB-amoxicillin	Wound dressing scaffold	2023	[207]
PHBV/Av; PHBV/Ho	Wound healing scaffold in murine wound	2025	[208]
OLE/PHBV; OLE/(PHB/PHOHD)	Scaffold for wound healing and tissue regeneration with antibacterial activity	2021	[209]
PHO-Bio-AgNPs	Bioactive film for wound healing and MRSA treatment (MRSA: methicillin-resistant *Staphylococcus aureus*)	2023	[210]
P(3HB-co-4HB)	Degradable low-crystalline system usable in wound healing process	2022	[211]
P(3HB)/P(3HO-co-3HD)-AgNPs	System for wound healing applications	2021	[212]
PHB; ε-PLL	Antimicrobial nonwovens for single applications such as medical gauze	2021	[213]
PHBV	Production of sustainable in vitro models by using industrial by-products.	2022	[214]
PHBH/CNCs	Bio-based and bioresorbable composite material for wound dressing applications	2023	[215]
P34HB/CIP/DMOG	Versatile wound dressing with effective stimulation of angiogenesis and antibacterial activity	2022	[216]
Fe_3_O_4_/RGO-g-PHBV	Composite porous scaffold with antimicrobial, biocompatible and biodegradable for fibroblast cell infiltration and proliferation	2016	[217]
PHBV/nCeO_2_	Wound dressing system to enhance cell proliferation and promote healing in diabetic wounds	2019	[218]

* Abbreviations: PHB/Collagen refers to poly(3-hydroxybutyrate) combined with collagen, while PHB-amoxicillin denotes poly(3-hydroxybutyrate) loaded with the antibiotic amoxicillin. PHBV/Av and PHBV/Ho represent poly(3-hydroxybutyrate-co-3-hydroxyvalerate) combined with Aloe vera extract or Houttuynia cordata extract, respectively. OLE/PHBV and OLE/(PHB/PHOHD) refer to olive leaf extract incorporated into PHBV or into a blend of poly(3-hydroxybutyrate) and poly(3-hydroxyoctanoate-co-3-hydroxydecanoate). PHO-Bio-AgNPs denotes polyhydroxyoctanoate containing bio-synthesized silver nanoparticles. P(3HB-co-4HB) represents poly(3-hydroxybutyrate-co-4-hydroxybutyrate). P(3HB)/P(3HO-co-3HD)-AgNPs refers to poly(3-hydroxybutyrate) combined with poly(3-hydroxyoctanoate-co-3-hydroxydecanoate) and silver nanoparticles. PHB; ε-PLL indicates poly(3-hydroxybutyrate) with ε-poly-L-lysine. PHBV denotes poly(3-hydroxybutyrate-co-3-hydroxyvalerate). PHBH/CNCs refers to poly(3-hydroxybutyrate-co-3-hydroxyhexanoate) reinforced with cellulose nanocrystals. P34HB/CIP/DMOG denotes poly(3-hydroxybutyrate-co-4-hydroxybutyrate) loaded with ciprofloxacin and dimethyloxalylglycine. Fe_3_O_4_/RGO-g-PHBV represents magnetite and reduced graphene oxide grafted onto PHBV. PHBV/nCeO_2_ refers to poly(3-hydroxybutyrate-co-3-hydroxyvalerate) combined with nanoceria (nanocrystalline cerium oxide).

**Table 10:** Nerve regeneration using PHAs.

Biopolymer *	Application	Year of Publication	References
PHA-(Nerve guidance conduits) PHA-NCGs	Peripheral nerve regeneration of the median nerve of female Wistar rats	2021	[246]
PHB/Fe_3_O_4_-citric acid (PHB/Fe_3_O_4_-CA)	Magnetic nerve guidance conduit (NGC) as implants for regeneration of peripheral nerves.	2024	[247]
PLA/PHA	Applications such as stents, NGCs, bone scaffolds	2023	[167]
MCL-PHA/PCL	Synthetic scaffold as substitute autologous nerve grafting	2021	[248]
P(3HO)/P(3HB)	Intraluminal aligned fiber guidance scaffold	2023	[249]
P(3HO-co-3HD)	System for nerve regeneration and clinical application in peripheral nerve repair	2023	[164]
PHB/chitosan-hMSC-bm	Artificial guide for nerve regeneration	2018	[250]
PDLLA/PHBHHx	System for nerve regeneration	2006	[251]
PHB/chitosan	Scaffold for nerve tissue engineering applications	2019	[252]
PHB	Nerve grafts for the clinical repair of traumatic nerve injuries	2015	[234]

* Abbreviations: PHA (nerve guidance conduits) refers to polyhydroxyalkanoate-based nerve guidance conduits, while PHA-NCGs denotes polyhydroxyalkanoate nerve conduit grafts. PHB/Fe_3_O_4_-citric acid (PHB/Fe_3_O_4_-CA) represents poly(3-hydroxybutyrate) combined with magnetite (Fe_3_O_4_) nanoparticles functionalized with citric acid. PLA/PHA indicates a composite of polylactic acid and polyhydroxyalkanoate. MCL-PHA/PCL refers to medium-chain-length polyhydroxyalkanoates blended with polycaprolactone. P(3HO)/P(3HB) denotes a combination of poly(3-hydroxyoctanoate) and poly(3-hydroxybutyrate), while P(3HO-co-3HD) represents poly(3-hydroxyoctanoate-co-3-hydroxydecanoate). PHB/chitosan-hMSC-bm refers to poly(3-hydroxybutyrate) with chitosan and human bone-marrow-derived mesenchymal stem cells. PDLLA/PHBHHx denotes poly(D,L-lactic acid) combined with poly(3-hydroxybutyrate-co-3-hydroxyhexanoate). PHB/chitosan represents a composite of poly(3-hydroxybutyrate) and chitosan, while PHB alone refers to poly(3-hydroxybutyrate).

**Table 11:** Different PHAs used as drug carriers.

PHA *	Drug/Model Drug Delivered	Target Tissues	Applications	Administration Routes	References
PHB (nanoparticles)	Sorafenib, Doxurubicin	Treatment of primer liver tumors	Anticancer agents release systems	Human blood plasma	[291]
P(3HB-co-4HB) (nanoparticles)	Docetaxel (DCX)	Treatment of lung tumors	Anticancer agents release systems	Dialysis membrane method	[292]
PHBV (nanoparticles)	Paclitaxel (PTX)	Treatment of liver tumors	Chemotherapeutic agent release system	Injection into the caudal vein	[293]
PHA (nanoparticles)	Paclitaxel (PTX)	Treatment of cervical cancer	Drug delivery system	In vitro study on HeLa cells	[294]
PHB/polyamina-NiO (nanoparticles)	Norfloxacin (NF)	Treatment of colorectal cancer	Antitumor and antibacterial delivery system	In vitro study on HepG-2 and WISH cell lines	[295]
PHBV-PEG (nanoparticles)	PT2399	Treatment of disk degeneration	Drug delivery system	Puncture injection	[296]
PHO/HAp	curcumin	Hard-tissue engineering applications	Drug delivery system	In vitro study	[297]
PHBV/ALG	insulin	Diabetes treatment	Hydrogels for long-acting insulin administration	Injection or infusion	[298]
PHB/CS	Doxorubicin, Indomethacin	Treatment of lung cancer	Aerogels for drug delivery	Injection	[299]
HA/jeffamine + PHBV/PLGA NPs	Teriparatide	Treatment of osteoporosis	Hydrogel for drug delivery system	Subcutaneous injection	[300]
PHB-co-PEI (nanoparticles)	miR-128	Treatment of brain cancer	Gene delivery system	In vitro delivery on U87 cells	[301]

* Abbreviations: PHB (nanoparticles) denotes poly(3-hydroxybutyrate) formulated as nanoparticles. P(3HB-co-4HB) (nanoparticles) represents poly(3-hydroxybutyrate-co-4-hydroxybutyrate) in nanoparticle form, and PHBV (nanoparticles) refers to poly(3-hydroxybutyrate-co-3-hydroxyvalerate) nanoparticles. PHA (nanoparticles) denotes polyhydroxyalkanoate nanoparticles. PHB/polyamina-NiO (nanoparticles) describes poly(3-hydroxybutyrate) combined with polyamine-modified nickel oxide nanoparticles. PHBV-PEG (nanoparticles) refers to poly(3-hydroxybutyrate-co-3-hydroxyvalerate) modified with polyethylene glycol in nanoparticle form. PHO/HAp denotes polyhydroxyoctanoate combined with hydroxyapatite. PHBV/ALG represents poly(3-hydroxybutyrate-co-3-hydroxyvalerate) blended with alginate, while PHB/CS refers to poly(3-hydroxybutyrate) combined with chitosan. HA/jeffamine + PHBV/PLGA NPs indicates hydroxyapatite functionalized with jeffamine alongside PHBV/poly(lactic-co-glycolic acid) nanoparticles. PHB-co-PEI (nanoparticle) denotes poly(3-hydroxybutyrate) grafted with polyethyleneimine in nanoparticle form.

## Data Availability

This study is a review article and does not report any new data. All data discussed are cited from previously published sources.
